# Fasting Upregulates *npy, agrp*, and *ghsr* Without Increasing Ghrelin Levels in Zebrafish (*Danio rerio*) Larvae

**DOI:** 10.3389/fphys.2018.01901

**Published:** 2019-01-24

**Authors:** Rafael Opazo, Francisca Plaza-Parrochia, Gustavo R. Cardoso dos Santos, Gabriel R. A. Carneiro, Vinicius F. Sardela, Jaime Romero, Luis Valladares

**Affiliations:** ^1^Laboratorio de Biotecnología INTA, Universidad de Chile, Santiago, Chile; ^2^Laboratorio de Endocrinología y Biología de la Reproducción, Hospital Clínico, Universidad de Chile, Santiago, Chile; ^3^Laboratorio de Pesquisa, Desenvolvimento e Inovação (LPDI-LADETEC), Instituto de Química, Universidade Federal do Rio de Janeiro, Rio de Janeiro, Brazil; ^4^Laboratorio de Hormonas y Receptores INTA, Universidad de Chile, Santiago, Chile

**Keywords:** ghrelin, fish larvae, feed intake regulation, neuropeptide Y, agouti-related peptide

## Abstract

Food intake in fish and mammals is orchestrated by hypothalamic crosstalk between orexigenic (food intake stimulation) and anorexigenic (food intake inhibition) signals. Some of these signals are released by peripheral tissues that are associated with energy homeostasis or nutrient availability. During the fish larva stage, orexigenic stimulation plays a critical role in individual viability. The goal of this study was to assess the mRNA levels of the main neuropeptides involved in food intake regulation (*npy, agrp, carppt*, and *pomc*), in concert with the mRNA levels and peptide levels of ghrelin, under a fasting intervention at the larval stage in zebrafish (*Danio rerio*). Prior to the fasting intervention, the zebrafish larva cohort was reared for 20 days post fertilization (dpf) and then randomly divided into two groups of 20 individuals. One group was subjected to a fasting intervention for 5 days (fasted group), and the other group was fed normally (fed group); this experimental protocol was performed twice independently. At the end of the fasting period, individuals from each experimental group were divided into different analysis groups, for evaluations such as relative gene expression, immunohistochemistry, and liquid chromatography coupled to nano high-resolution mass spectrometry (nLC-HRMS) analyses. The relative expression levels of the following genes were assessed: neuropeptide Y (*npy*), agouti-related peptide (*agrp*), proopiomelanocortin (*pomc*), cocaine and amphetamine-regulated transcript (*cartpt*), ghrelin (*ghrl*), ghrelin O-acyltransferase (*mboat4*), growth hormone secretagogue receptor (*ghsr*), and glucokinase (*gck*). In the fasted group, significant upregulation of orexigenic peptides (*npy* – *agrp*) and *ghsr* was observed, which was associated with significant downregulation of *gck*. The anorexigenic peptides (*pomc* and *cartpt*) did not show any significant modulation between the groups, similar to *mboat4*. Contrary to what was expected, the relative mRNA upregulation of the orexigenic peptides observed in the fasted experimental group could not be associated with significant ghrelin modulation as assessed by three different approaches: qPCR (relative gene expression of ghrelin), nLC-HRMS (des-acyl-ghrelin levels), and immunohistochemistry (integrated optical density of prepropeptides in intestinal and hepatopancreas tissues). Our results demonstrate that zebrafish larvae at 25 dpf exhibit suitable modulation of the relative mRNA levels of orexigenic peptides (*npy* and *agrp*) in response to fasting intervention; nevertheless, ghrelin was not coregulated by fasting. Therefore, it can be suggested that ghrelin is not an essential peptide for an increase in appetite in the zebrafish larva stage. These results give rise to new questions about food intake regulation factors in the early stages of fish.

## Introduction

Food intake in mammals and fish is regulated by the hypothalamus. In mammals, the key nuclei associated with the key appetite-regulating role are the arcuate nucleus neurons, which coordinate regulation in the central nervous system (CNS) by projecting their axons to other main hypothalamic and extrahypothalamic areas (Cansell et al., 2012). However, the hypothalamus of fish presents neuroanatomical differences from that of mammals (Biran et al., 2015), with the dorsal area of the periventricular and lateral hypothalamus being the region characterized by the same neuropeptides as the arcuate nucleus (Jeong et al., 2018). These appetite-regulating neurons can be divided into two populations: neurons that coexpress neuropeptide Y (Npy) and agouti-related peptide (*agrp*), which are orexigenic (food intake stimulatory) neuropeptides, and neurons that coexpress proopiomelanocortin (*pomc*) and the cocaine and amphetamine-regulated transcript (*cart*), which are anorexigenic (food intake inhibitory) neuropeptides (Sobrino Crespo et al., 2014; Chu et al., 2015). Food intake regulation is also orchestrated by peripheral signals, such as peptides, nutrients, or nerve pathways, which inform the CNS about energy homeostasis and nutrient availability in the body (Anand and Brobeck, 1951; Gorissen et al., 2006; Moran and Ladenheim, 2016). Peripheral peptides exert their effects mainly by binding hypothalamic neuronal receptors. In fish, food intake regulation shares important similarities with that in mammals (Volkoff and Peter, 2006; Volkoff et al., 2009); the lateral tuberal nucleus located in the ventral hypothalamus is the teleost homolog of the arcuate nucleus (Cerdá-Reverter et al., 2003), which is integrated by key appetite-regulating neuronal populations that coexpress the above neuropeptides (Volkoff, 2016; Rønnestad et al., 2017).

A large group of food intake regulation factors from peripheral tissues has been described, and these factors exhibit specific receptors in key appetite-regulating hypothalamic neurons (Volkoff, 2016; Prinz and Stengel, 2017). Among these peripheral factors, only ghrelin has been described as exerting orexigenic effects (Higgins et al., 2007; Valassi et al., 2008; Volkoff, 2016). Ghrelin is a pleiotropic peptide that is mainly produced by the endocrine X/A-like cells of the stomach and intestine (Kojima et al., 1999; Nakazato et al., 2001; Dimaraki and Jaffe, 2006). In mammals, ghrelin is a 28-amino acid peptide, but in fish, it has been reported to differ by species (ranging from 20 to 27 amino acids) (Kaiya et al., 2011). Ghrelin is a complex peptide because it exhibits splice variants in mammals and fish (Unniappan and Peter, 2005) and requires a unique acyl modification on the third position serine for it to exhibit any physiological and biological activity. This modification is catalyzed in the endoplasmic reticulum by ghrelin O-acyltransferase (Mboat4) (Unniappan and Peter, 2005; Yang et al., 2008; Müller et al., 2015).

Ghrelin’s physiological effects are exerted through its specific receptor, the growth hormone secretagogue receptor (*ghsr*) (Unniappan and Peter, 2005; Kaiya et al., 2008; Müller et al., 2015). The key appetite-regulating neuronal populations in the arcuate nucleus express Ghsr, although it has also been described as being present in the paraventricular nucleus and ventromedial nucleus (Guan et al., 1997). Exogenous administration of ghrelin increases food intake in humans, rodents and fish (Kang et al., 2011; Müller et al., 2015; Alamri et al., 2016).

The regulation of the relative gene expression of ghrelin and its secretion is associated with different factors, such as nutrient ingestion (mainly glucose and fatty acids), insulin, glucagon, leptin, growth hormone (Gh), insulin-like growth factor 1 (Igf-1), somatostatin, and estrogen. However, nutrient intake is the most important factor that decreases the levels of ghrelin expression and secretion (Yin et al., 2009). In mammals and fish, circulating levels of ghrelin rise in association with preprandial periods or fasting intervention, and they decrease in postprandial periods (Drazen et al., 2006; Miura et al., 2006, 2007; Picha et al., 2009; Hevrøy et al., 2011; Kang et al., 2011; Müller et al., 2015; Alamri et al., 2016). In mammals, when ghrelin binds to its receptor (Ghsr), it causes an increase in the mRNA levels of the orexigenic peptides *npy* and *agrp* in the neurons of the arcuate nucleus (Willesen et al., 1999; Kamegai et al., 2001; Nakazato et al., 2001; Miura et al., 2006).

The fish larval stage is characterized by a metamorphosis process in which anatomical and physiological changes occur (Rønnestad and Morais, 2008). Orexigenic stimulation is critical for improving the survival of fish larvae because this stage is characterized by high energy and nutrient demands (Hamre et al., 2013). This study’s goal was to assess hypothalamic neuropeptides and peripheral ghrelin under a fasting intervention (challenge) and thereby enhance our knowledge of food intake regulation in this critical fish stage.

## Materials and Methods

### Zebrafish (*Danio rerio*) Husbandry and Fasting State Intervention

**FIGURE 1:**
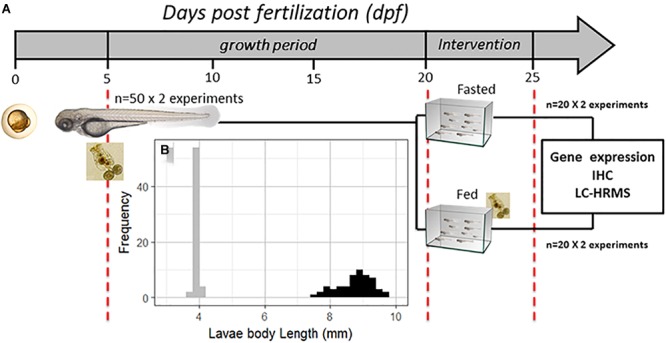
**(A)** Schematic diagram of the experimental design; the scale is indicated in days post fertilization (dpf); at 5 dpf, the larvae received the first feeding with rotifers (*Brachionus plicatilis*) at a rate of 800 per larva per day and were reared for 15 days. Then, at 20 dpf, the larvae were divided into two groups in different glass tanks. Over the next 5 days, one group received food (rotifers) continuously (fed group), and another group did not (fasted group). **(B)** Frequency histogram of larval body length (mm) at 5 dpf (gray, *n* = 100) and 20 dpf (black, *n* = 80). IHC, immunohistochemistry; Frequency, number of larvae for each body length range; nLC-HRMS, nano liquid chromatography with high-resolution mass spectrometry.

Wild-type zebrafish larvae were reared in a 2-L glass tank with E2 methylene blue medium (Westerfield, 2000) at 28°C, under a controlled light/dark cycle (14 L/10D); 30% of the water was changed every day for 20 days. Larval feeding began at 5 dpf, supplying rotifers (*Brachionus plicatilis*) at a rate of 800 rotifers per larva per day (Opazo et al., 2017a); the rotifer biomass composition (dry weight) was as follows: protein = 55.7%, lipid = 19.5%, carbohydrate = 19.0%, and ash = 5.8%. At 20 dpf, the larval cohort was randomly divided into two groups in different glass tanks (*n* = 20 per experimental group). The fasting intervention was conducted for 5 days; one group of larvae was not fed for 5 days (fasted group), and the other group was maintained on a normal rotifer feeding schedule (fed group), as shown in Figure 1A. This experimental protocol was performed using two independent replicates. After 5 days of fasting, larvae from both groups were randomly distributed to three different analysis groups, for relative gene expression, immunohistochemistry and nanoliquid chromatography with high-resolution mass spectrometry (nLC-HRMS) analyses. For relative gene expression analysis, 5 individuals were selected from each group and for each experimental replicate (*n* = 10 per experimental group); for immunohistochemistry, 3 individuals were selected from each group and for each experimental replicate (*n* = 6 per experimental group); and for nLC-HRMS, 4 individuals were selected from each group and for each experimental replicate (*n* = 8 per experimental group). Additionally, all larvae were measured at 5, 20, and 25 dpf to establish the larvae cohort growth rate or Pearson’s correlation between the relative mRNA levels of the genes and larval body length. The body length measurements were conducted with a stereoscopic microscope using Motic^®^ Images Plus 2.0 ML software according to the protocol proposed by Parichy et al. (2009). Body length was measured from the snout to the end of the caudal fin, and the larvae were first anesthetized with tricaine methanesulfonate. This study was conducted in strict accordance with the recommendations of the European Convention for the Protection of Vertebrate Animals used for Experimental and other Scientific Purposes (Council of Europe No 123, Strasbourg, 1985) and the Guide for the Care and Use of Laboratory Animals from the National Institutes of Health. The protocol was approved by the Committee on the Ethics of Animal Experiments of INTA Universidad de Chile.

### Relative Gene Expression Analysis

Each individual (larva) was placed in a 1.7-mL microcentrifuge tube and euthanized by freezing to a temperature of -80°C in liquid nitrogen. Total RNA was isolated using 800 μl of the TriPure^®^ reagent (cat.#11667165001 Roche, Mannheim, Germany), according to the manufacturer’s instructions. RNA quality and quantity were assessed by spectrophotometry at 260 and 280 nm (NanoDrop^®^) and via fluorometric quantitation (Qubit^®^). The samples were treated with RQ1 RNase-Free DNase (cat. #M6101, Promega, Madison, WI, United States) to degrade genomic DNA, and the absence of genomic DNA was confirmed through qPCR of the treated RNA as a DNA control. First-strand cDNA synthesis was performed using the ImProm-II^TM^ Reverse Transcription System (cat. #A3800, Promega, Madison, WI, United States), according to the manufacturer’s instructions. The total RNA was combined with 0.5 μg/reaction of oligo(dT)15 primer (cat. #C1101, Promega) for a final volume of 5 μl and then incubated at 70°C for 5 min. Next, 15 μl of the transcription mixture (4.6 μl ImProm-II^TM^ 5X Reaction Buffer, 2.25 mM MgCl2, 0.5 mM each dNTP and Recombinant RNasin^®^ Ribonuclease Inhibitor (cat. #N2511, Promega, Madison, WI, United States) in a volume of 20 μl) and 1 U of ImProm-II^TM^ Reverse Transcriptase were added. Following the addition of the transcription mixture, the reaction was maintained at 25°C for 5 min and then at 42°C for 60 min. The reverse transcription reactions were stopped by heating the mixture to 70°C for 15 min.

The gene-specific oligonucleotide primers for ghrelin (*ghrl*), ghrelin O-acyltransferase (*mboat4*), *ghsr, agrp*1, neuropeptide Y (*npy*), *pomc1, cartpt1* and glucokinase (*gck*) were developed using Primer 3 (Untergasser et al., 2012) and are listed in Table 1. To normalize cDNA loading, all samples were run in parallel using the housekeeping gene elongation factor I-alpha (*ef1α*) as the reference gene. This reference gene has been assessed and recommended for fasting intervention or nutritional deprivation studies in fish (McCurley and Callard, 2008; MacDonald and Volkoff, 2009; Valen et al., 2011; Hatef et al., 2015; Tian et al., 2015). The relative mRNA expression levels of both the target genes and the reference gene were quantified through real-time PCR analysis with AriaMx^®^ (Agilent Technologies). The amplification of specific PCR products was detected using FastStart Essential DNA Green Master^®^ (cat. #0640272001 Roche, Mannheim, Germany), according to the manufacturer’s instructions. All cDNA samples from each individual were analyzed in duplicate. The amplification protocol was as follows: one initial step at 95°C for 10 min (denaturation and enzyme activation), followed by 45 cycles at 95°C for 10 s, 60°C for 5 s and 72°C for 15 s. After amplification, a melting curve analysis was performed over a range of 50–95°C to verify that a single PCR product was generated at the end of the assay.

**Table 1:** Primers used for the quantification of the mRNA expression by real-time PCR.

Target Gene	Gene Symbol	GenBank Accession no.	Position	Product length (bp)	Sequence of Primers
Agouti-related peptide	*agrp*	XM_009303347	295–443	148	(F) ATGAGGATCTGGGCAAAGCTGT
					(R) GAAGGCCTTAAAGAAGCGGCAGTA
Cocaine and amphetamine-regulated transcript	*cartpt1*	AW280905.1	1–75	75	(F) TAGACACGATGGTCGGAGAGC
					(R) CACAGCGAACCAGCAGCATT
Elongation factor 1 alpha	*ef1-α*	NM_131263.1	1414–1516	103	(F) GTGCTGGCAAGGTCACAAAG
					(R) AGAGGTTGGGAAGAACACGC
Glucokinase	*gck*	NM_001045385.2	952–1119	168	(F) ACGAGAAGCTGATTGGTGGG
					(R) TGTCCCCTGTGTCACTCTCA
Ghrelin	*ghrl*	NM_001083872.1	23–158	136	(F) GCAGCATGTTTCTGCTCCTG
					(R) TCAGCAGCTTCTCTTCTGCC
Ghrelin O-acyltransferase	*mboat4*	NM_001122944.1	988–1230	242	(F) CCTGGGCAGATTCTGGGTTT
					(R) CAGAGCACTCAGCAGTGGAA
Growth hormone secretagogue receptor	*ghsr*	NM_001146272.1	439–589	151	(F) GTAGTCGTGACCAAGGGTCG
					(R) ATTCCGTCGCTTTGCATTCG
neuropeptide Y	*npy*	NM_131074	360–551	191	(F) CTGTGATGTCCATGTGTCCTTCTG
					(R) GAGCCTAAAGAGCGCACATTGA
Pro-opiomelanocortin	*pomc*	NM_131074	220–383	163	(F) CAGAGTCTGAGCTTGGGTTTGCTT
					(R) ACTTTTACCGGTCTGCGTTTGC

The relative expression levels of the genes were calculated with the method described by Pfaffl (2001). The reference group for the Pfaffl formula was obtained from 10 other larvae, which were randomly selected at 20 dpf and processed in the same way as for the above protocols. PCR primer efficiency was calculated for the fluorescence curve of each gene in LinRegPCR 12.18 software, and the efficiency rates for the transcripts were as follows: 1.96 for *ghrl*, 1.95 for *mboat4*, 1.98 for *ghsr*, 1.98 for *agrp1*, 1.9 for *npy*, 1.98 for *pomc1*, 1.96 for *cartpt*, 1.98 for *gck* and 1.9 for *ef1α* over the entire quantification range.

### Ghrelin Immunohistochemical Analyses

A rabbit polyclonal anti-zebrafish ghrelin/obestatin prepropeptide primary antibody (cat. #LS-C209887, LifeSpan BioSciences Inc., Seattle, WA, United States) and a sheep anti-rabbit polyclonal antibody (cat. #LS-C181152 LifeSpan BioSciences Inc., Seattle, WA, United States) were used for immunohistochemistry. Antigen labeling was conducted with diaminobenzidine (DAB) (cat. #K3468 DAKO, United States). The staining procedure was performed as previously described (Plaza et al., 2010). In brief, the whole individual was embedded in paraffin. Then, 5-μm sections of formalin-fixed paraffin-embedded samples were obtained. Thereafter, the tissue sections were deparaffinized in xylene and gradually hydrated through an alcohol gradient. The sections were incubated in an antigen retrieval solution (10 mmol L^-1^ citrate buffer, pH 9.5) at 100°C for 20 min. The sections were subsequently incubated in 3% hydrogen peroxide for 15 min to prevent any endogenous peroxidase activity. Non-specific antibody binding was prevented by incubation in 4% phosphate-buffered saline with bovine serum albumin (PBS–BSA) for 1 h. The primary ghrelin antibody (1:100) was applied to the samples, followed by incubation overnight at 4°C. The negative controls were analyzed in adjacent sections incubated without the primary antibody. The secondary antibody was biotinylated sheep anti-rabbit immunoglobulin (1:300) and was incubated for 30 min at 37°C. The reaction was developed using the streptavidin-peroxidase system; DAB was used as the chromogen, with incubation for 30 s, and counterstaining with hematoxylin was then performed. The immunostaining protocol conducted in this study was standardized prior to the experiment to determine the appropriate incubation time, concentrations of the antibodies, and time of DAB staining. Integrated optical density (IOD) analysis was performed according to the method of Paizs et al. (2009) in 1000X microscopic magnification equivalent areas of the hepatic and intestinal epithelia, using ten images replicates per slide sample; this analysis was performed with Image-Pro Plus 6.2 software, and IOD values are expressed in arbitrary units.

### Liquid Chromatography Coupled to Nano High-Resolution Mass Spectrometry (nLC-HRMS)

Larval tissue preparation for proteomic analysis was conducted via this protocol. Each larva (*n* = 8 per group) ≈10–13 mg, was placed in a 1.7-mL microcentrifuge tube and was then snap-frozen in liquid nitrogen (LN_2_). Next, 100 μl of RIPA buffer was added (Peach et al., 2015), and the larvae were incubated with RIPA buffer for 5 min in ice. After the incubation, the larval tissue was homogenized with a microtube sample pestle. The homogenized tissue was incubated in RIPA buffer for 20 min in ice. Then, the homogenized tissue was centrifuged for 20 min at 15,000 *g* at 4°C, the supernatant containing soluble proteins was transferred to a new tube, after which the centrifugation process was repeated under the same conditions. After the second centrifugation, an aliquot was taken from each larval supernatant for quantification with a BCA Protein Assay Kit (cat.#23225, Pierce Thermo scientific^®^, Rockford, IL, United States) and stored at -80°C.

Prior to chromatographic injection, human ghrelin (5 ng mL^-1^) (cat. #AS-24157, AnaSpect, Fremont, CA, United States) was added to each sample as an internal standard. Thereafter, the protein samples were cleaned with a Detergent Removal Spin Column (cat. #87777,Pierce Thermo scientific^®^, Rockford, IL, United States) and precipitated via centrifugation for 15 min at 13,000 *g* in 100 μl of liquid chromatography acetonitrile (ACN; cat. #100030, Merck^®^, Darmstadt, Germany) and 50 μl of 1% glacial acetic acid (cat. #101830, Merck^®^) per 25 μl protein sample. The liquid in the protein supernatant was then evaporated by a Speedvac at 45°C and resuspended in 2% glacial acetic acid.

The reagents used for testing were ACN (MS grade), formic acid (p.a.) and MS grade water (Tedia Brazil, São Paulo, Brazil). Acetic acid (glacial) and sodium hydroxide (p.a) (#100063, Merck^®^, Darmstadt, Germany). A 20-μl aliquot of each sample was introduced to an Ultimate 3000 nano Liquid-Chromatography system (ThermoScientific, Bremen, Germany) using a 300 μm × 5 mm C18 PepMap 100 μ-Precolumn (5 μm, 100 Å) and an analytical PicoChip column (REPROSIL-Pur C18-AQ 3 μm 120 Å; 105 mm). Phase A consisted of MilliQ water containing 0.2% formic acid, and phase B consisted of ACN containing 0.2% of formic acid. A gradient of 15–90% mobile phase B over 65 min at a flow rate of 300 nL min^-1^ was applied.

The tuning conditions for peptide ionization included a capillary temperature of 380°C, spray voltage of 3.9 kV, positive polarity, and S-lens RF level of 80. Mass spectra were acquired with a Quadrupole-Orbitrap mass analyzer (QExactive Plus, ThermoScientific, Bremen, Germany) using FullScan-Target-SIM-ddMS2and PRM mode acquisitions.

For Fullscan-Target-SIM acquisitions, the instrument was configured as follows: (a) FullScan acquisition, 17,500 resolution; AGC target 1e5; maximum IT 50 ms; scan range 400–2000 *m/z*. (b) Target-SIM, microscans 1; 70,000 resolution; AGC target 5e5; maximum IT 60 ms; loop count 4; MSX count 4; isolation window 4.0 *m/z*; scan range 560.00–564.00 e 526.00–530.00 *m/z* e. The area of the spectra and the peak overlay were determined and integrated using Trace Finder TM 3.2 software. We obtained the chemical formula of zebrafish proghrelin from the UniProt database (D0EW40), although the specific sequence of the des-acyl-ghrelin peptide [19 aa (28–46)] proposed by Amole and Unniappan (2009) (gtsflsptqkpqgrrpprv) was selected. Then, using Xcalibur software (Thermofisher), the molecular mass and isotopic envelopes were calculated for zebrafish des-acyl-ghrelin and human acyl-ghrelin. Additionally, a comparison of these human ghrelin data with the results obtained with an FWHM of 70,000 resulted in identification with an error of less than 3 ppm for this structure in the chromatographic peak. Therefore, we assume that the peak characterized the target peptide.

### Statistical Evaluation

The differences in relative gene expression levels between the larval groups were analyzed with the Mann–Whitney test using R-3.1.2 for Windows 49; *p*-values < 0.05 were considered significant. The normality of the distribution of the data was assessed via the Shapiro–Wilk test, and some data did not show a normal distribution; hence, the differences observed in the relative gene expression, immunohistochemistry and nLC-HRMS analyses were performed via the Mann–Whitney test using R-3.4.1 statistical software (De Neve et al., 2013); *p*-values less than 0.05 indicated significant differences. Additionally, the Bartlett test was used to analyze the homoscedasticity between the data from the experimental groups. Pearson’s correlation among gene mRNA levels and larval body length was performed using R-3.4.1 statistical software with the Hmisc package.

## Results

To establish and assess zebrafish larval growth, body length was first measured at 5 dpf (first day of exogenous feeding), showing an average of 4.0 ± 0.06 mm. Then, at 20 dpf (before the fasting intervention began), larval body length was measurement again, showing an average of 8.7 ± 0.64 mm; see Figure 1B. The mortality rate before the fasting intervention was 2% in both zebrafish larva groups. Larval mortality is a factor that can limit the number of intervention days at this stage.

**FIGURE 2:**
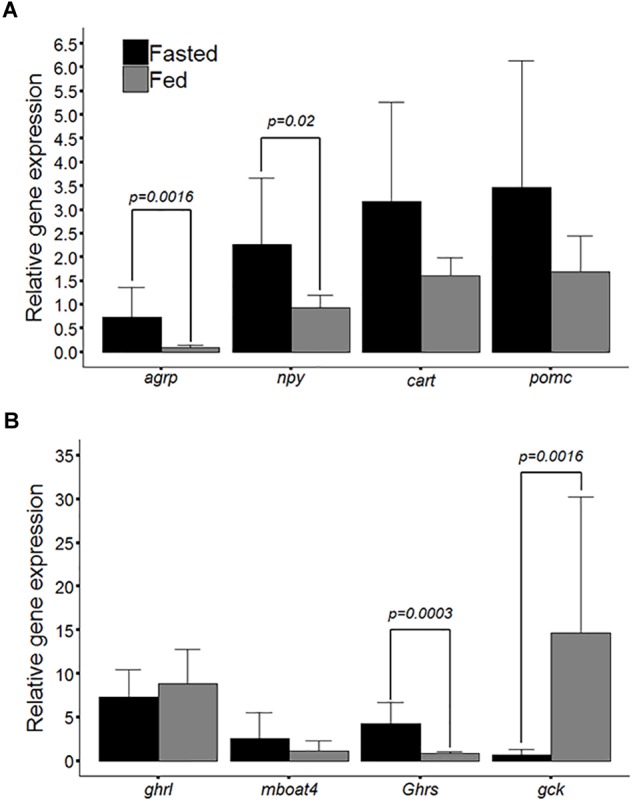
**(A)** Bar plot of relative mRNA levels for the agouti-related peptide (*agrp*), cocaine and amphetamine-regulated transcript (*cartpt*), neuropeptide Y (*npy*) and proopiomelanocortin (*pomc*) between larval zebrafish groups (fasted and fed). **(B)** Bar plot of relative mRNA levels for ghrelin (*ghrl*), ghrelin O-acyltransferase (*mboat4*), growth hormone secretagogue receptor (*ghsr*) and glucokinase (*gck*). The values are means ± SD (*n* = 10), and significant differences (Mann-Whitney *U*-test) are denoted by *P*-values.

The relative gene expression observed between the fasted and fed groups is presented in Figures 2A,B. Figure 2A shows the relative gene expression of the *npy, agrp, cartpt*, and *pomc* peptides, the differences between the groups in terms of the mRNA levels observed for *agrp* and *npy* were statistically significant (*p* = 0.0016 and *p* = 0.02, respectively), but those of *cartpt* and *pomc* were not significantly different (*p* = 0.24 and *p* = 0.21, respectively). Figure 2B shows the relative gene expression observed for ghrelin (*ghrl*), *ghsr, mboat4*, and *gck*. Statistically significant differences were observed between the experimental groups for *gck* and *ghsr* (*p* = 0.001 and *p* = 0.0003, respectively). Notwithstanding these modulations of the relative gene expression of orexigenic peptides, the differences in *ghrl* and *mboat4* mRNA levels between the experimental groups were not significantly different (*p* = 0.39 and *p* = 0.52, respectively). Pearson’s correlation analysis between genes and larval body length showed that the relative gene expression of *ghrl* presented the highest significant positive correlation with larval body length of 0.61 (*p* = 0.0046); conversely, *agrp* presented the lowest significant negative correlation, of -0.63 (*p* = 0.0032); see Table 2.

**Table 2:** Pearson correlation coefficients between mRNA levels and zebrafish larval body length.

	*ghrl*	*ghsr*	*gck*	*npy*	*pomc*	*mboat4*	*agrp*	*cartpt*
Larval body length	(0.61)	(–0.59)	0.19	–0.39	–0.29	–0.32	(–0.63)	–0.49

*()indicate significant correlations.*

**FIGURE 3:**
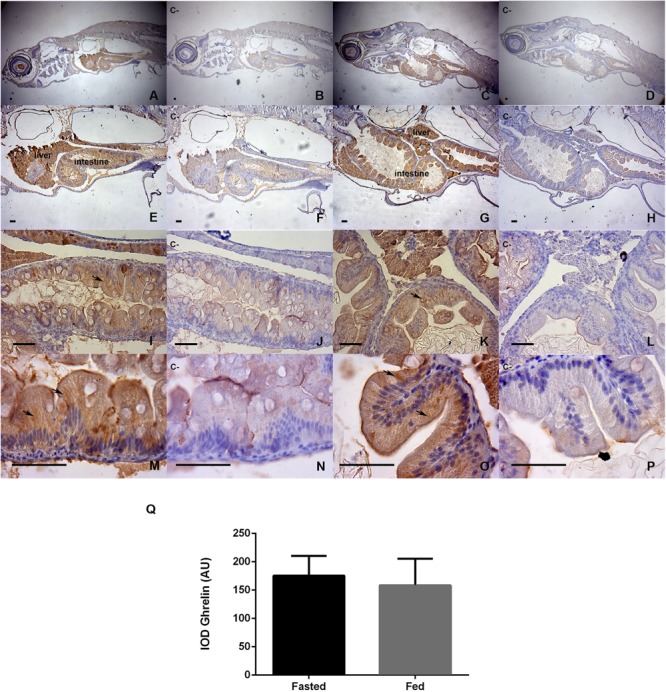
Immunohistochemical detection of ghrelin in larval zebrafish tissues in fasted and fed groups. The staining time in DAB was 30 s. Representative microphotographs were obtained at 40x magnification **(A–D)**, 100X **(E–H)**, 400X **(I–L)**, and 1000X **(M–P)**; the bars represent 50 μm. Fasted larvae are represented by photographs **(A,E,I,M)**, contrasted with their negative controls **(B,F,J,N)**. The fed larvae are represented by photographs **(C,G,K,O)**, contrasted with their negative controls **(D,H,L,P)**. Negative controls were analyzed in adjacent sections incubated without the primary antibody. The black arrows show the most intensely stained areas for the ghrelin prepropeptide, and the integrated optical density (IOD) was evaluated by the method of Paizs et al. (2009), in areas of hepatic and intestinal epithelia at 1000X microscopic magnification, using equivalent areas and ten images replicates per slide sample; the analyses was performed with Image-Pro Plus software. Bar plot **(Q)** show a semiquantification analysis for both study groups. Values are means ± SD (*n* = 6), the difference was not significant in the Mann-Whitney *U*-test.

The results of the ghrelin immunohistochemistry analysis are presented in Figure 3. Figures 3A,C (40X) show that both groups (fasted and fed) presented positive staining for the ghrelin prepropeptide in the hepatopancreas, intestine, retina, and gills. However, higher magnifications of 100X and 400X (Figures 3E,G,I,K) showed that the main staining areas were located in hepatopancreas and intestine tissues. The fasted group is represented by photographs Figures 3A,E,I,M, which are contrasted with their negative controls (Figures 3B,F,J,N). The fed group is represented by photographs Figures 3C,G,K,O, which are contrasted with their negative controls (Figures 3D,H,L,P). The staining was shown to be homogeneous and was mainly cytoplasmic, without nuclear immunostaining. The strongest staining was observed at the apex of intestinal cells [Figures 3M,O (1000X)]. For the semiquantitative analysis of ghrelin, as assessed by an IOD, no statistically significant differences in intestinal immunostaining were detected between the groups (Figure 3Q).

**FIGURE 4:**
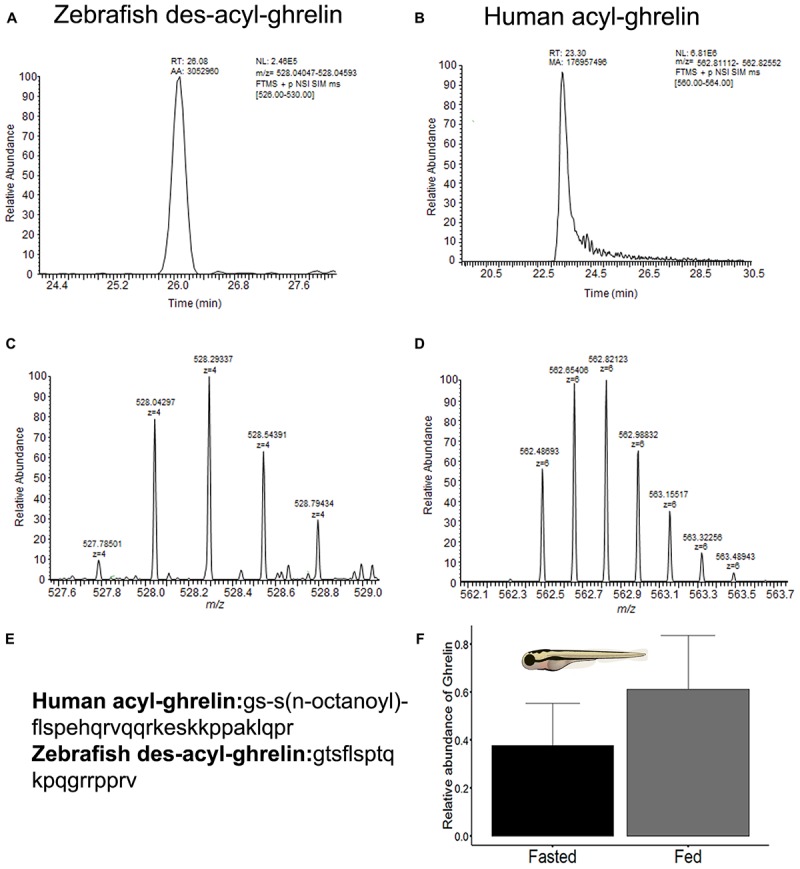
**(A)** Appearance of the chromatogram with the retention time peak observed for zebrafish des-acyl-ghrelin. **(B)** Appearance of the chromatogram with the retention time peak observed for human acyl-ghrelin (I.S). **(C)** Charge envelope of zebrafish des-acyl-ghrelin ionization obtained with a Quadrupole-Orbitrap nano high-resolution mass analyzer. **(D)** Charge envelop of human acyl-ghrelin ionization obtained with a Quadrupole-Orbitrap nano high-resolution mass analyzer. **(E)** Amino acid sequence of the mature zebrafish *(Danio rerio*) des-acyl-ghrelin peptide (NCBI accession number: NP_001077341.1) and the mature human (*Homo sapiens*) acyl-ghrelin peptide (NCBI accession number: NP_001289754.1). **(F)** Bar plot of the relative abundance of mature des-acyl-ghrelin levels between zebrafish larva experimental groups (fasted and fed). The values are means ± SD (*n* = 8), and the difference was not significant according to the Mann-Whitney *U*-test.

The results of nLC-HRMS for mature zebrafish des-acyl-ghrelin and human acyl-ghrelin are shown in Figures 4A–D. The sample clean-up procedure avoids the domination of mass spectra by detergents, which may occur even when present at low concentrations, owing to their ready ionizability and high abundance compared to individual peptides. Therefore, low ion suppression and an absence of interference were achieved. Consequently, a high intensity of the chromatographic peak related to ghrelin detectability was observed (Figures 4A,B). In addition, human acyl-ghrelin was applied as an internal standard (I.S.) in both groups and was used to calculate the relative intensity of des-acyl-ghrelin in zebrafish larva samples. Although human mature ghrelin showed differences in its amino acids sequence in comparison with zebrafish mature ghrelin (Figure 4E), the homology between human and zebrafish ghrelin allows comparisons between signal intensity, ionization and control recovery and chromatographic behavior for all injections. The retention time, *m/z* and isotopic envelope for target peptides, zebrafish des-acyl-ghrelin, and I.S. were compared in Figures 4A–D. An *m/z* of 528.29337, a charge state of 4+ and a retention time of 26.08 min were observed for zebrafish des-acyl-ghrelin, while an *m/z* of 562.82123, a charge state of 6+ and a retention time of 23.30 were observed for human acyl-ghrelin. Therefore, it could be assumed that any influence of the samples on the detectability of the peptides would be similar and that the concentration was calculated correctly.

Finally, the difference in the ghrelin levels observed via nLC-HRMS was not statistically significant between the zebrafish larva groups *p* = 0.09 (Figure 4F), although the fed group showed a higher mean ghrelin level than the fasted group. Jointly, the nLC-HRMS data exhibited homogeneity of variance, as assessed by the Bartlett test (*p* = 0.6); hence, I.S. produce better data normalization.

## Discussion

The main goal of the present study was to assess the modulation of food intake signals in zebrafish larvae under a fasting intervention. The observed larval growth rate was consistent with the growth curves from other zebrafish larvae reported by Parichy et al. (2009) and Gómez-Requeni et al. (2010). Hence, the larvae were in a suitable condition before the fasting intervention began.

The fasting intervention downregulated the mRNA levels of *gck*, which is a key liver enzyme for glucose metabolism through catalyzing the phosphorylation of glucose to glucose-6-phosphate (Enes et al., 2009; Polakof et al., 2011). The downregulation of *gck* indicates that fasting in zebrafish larvae leads to glucose restriction. This result was similar to other fish studies in which fasting interventions have been shown to downregulate *gck* mRNA levels (Caseras et al., 2000; González-Alvarez et al., 2009; Panserat et al., 2014).

Additionally, the fasting intervention upregulated the orexigenic peptides *npy* and *agrp*, as expected, which has also been described in other fish studies, including those of Narnaware et al. (2000) in adult goldfish (*Carassius auratus*), Silverstein et al. (1998) in coho salmon (*Oncorhynchus kisutch*) and chinook salmon (*Oncorhynchus chinook*), Hosomi et al. (2014) in yellowtail (*Seriola quinqueradiata*), Agulleiro et al. (2014) in sea bass (*Dicentrarchus labrax*), Wei et al. (2014) in marinka prenantova (*Schizothorax prenanti*) and Song et al. (2003) in zebrafish (*Danio rerio*).

Anorexigenic peptides did not display any differences in mRNA levels between the larva groups. Other fish fasting studies that have assessed *pomc* mRNA levels, such as those of Takahashi et al. (2005) in barfin flounder (*Verasper moseri*), Cerdá-Reverter et al. (2003) in goldfish (*Carassius auratus*) and Song et al. (2003) in zebrafish (*Danio rerio*), agree with this observation. In contrast, two different responses of relative *cartpt* gene expression have been described in fish fasting studies. Some authors have observed downregulation of *cartpt* mRNA levels associated with their fasted groups (Volkoff and Peter, 2001; Kehoe and Volkoff, 2007; Kobayashi et al., 2008; Murashita et al., 2009; Nishio et al., 2012; Akash et al., 2014; Volkoff, 2014; Volkoff et al., 2017). However, others have not observes differences in the *cartpt* mRNA levels, similar to our study; these studies include those by MacDonald and Volkoff (2009) in winter flounder (*Pseudopleuronectes americanus*), Murashita et al. (2009) in medaka (*Oryzias latipes*), and Gomes et al. (2015) in *Hippoglossus hippoglossus* larvae.

Despite potential glucose or nutrient restriction due to the fasting intervention, the zebrafish larva groups (25 dpf) did not show any significant differences in ghrelin levels according to either of the adopted technical approaches. This result is contradictory to the results described in other zebrafish (*Danio rerio*) fasting studies, conducted in adults or juvenile individuals (Eom et al., 2013; Tian et al., 2015). Likewise, other fish studies have described upregulation of ghrelin mRNA levels under a 7-day of fasting challenge (Blanco et al., 2016; Zhou et al., 2016; Dar et al., 2018). Nevertheless, not all fish studies have described ghrelin mRNA modulation due to fasting challenge, such as the studies of Koven and Schulte (2012) in zebrafish (*Danio rerio*), Riley et al. (2008) in tilapia (*Oreochromis mossambicus*), and Xu and Volkoff (2009) in Atlantic cod (*Gadus morhua*). Different arguments have been suggested to explain these contradictory results, but ghrelin physiology in fish is in fact poorly understood and requires further functional studies (Jönsson, 2013). The relative gene expression results observed in this study between the experimental groups were confirmed by immunohistochemistry and nLC-HRMS, to clearly establish the absence of ghrelin modulation by fasting in zebrafish larvae. For appropriate interpretation of the results, especially considering the analysis of whole zebrafish larvae, is important consider that relative ghrelin gene expression is mainly observed in different regions of the intestine and other peripheral tissues, which account for more than 98% of ghrelin in the body; hence, brain ghrelin is insignificant in comparison with peripheral ghrelin in zebrafish (Eom et al., 2013).

Another interesting observation based on our results was that ghrelin mRNA levels showed a lower tendency in the fasted group than in the fed group, which was corroborated by nLC-HRMS analysis; this tendency was contrary to any possibility that ghrelin was stimulated by the fasting intervention. In this regard, although *mboat4* did not show significant differences between the experimental groups, its tendency was accord with the expectations.

In fish, the ghrelin receptor (Ghsr) is mainly distributed in the hypothalamus, with the highest levels being present in the anterior periventricular nucleus (Sánchez-Bretaño et al., 2015). The significant increment of *ghsr* in the fasted experimental group reinforces the idea that melanocortin system neurons are mature in zebrafish larvae. In this regard, Blanco et al. (2016) described upregulation of *ghsr* in goldfish (*Carassius auratus*) in the telencephalon and hypothalamus associated with a fasting intervention, in accord with our results. In mammals, Npy/Agrp neurons can express the Ghsr receptor and therefore can receive ghrelin orexigenic peripheral signaling (Willesen et al., 1999; Chen et al., 2004); hence, the exogenous administration of ghrelin increases *npy* and *agrp* mRNA levels in mammals. Acyl-ghrelin administration in goldfish (*Carassius auratus*) was shown to stimulate food intake, and this outcome was associated with upregulation of *npy*, which was blocked with an Npy Y1-receptor antagonist (BIBP-3226) (Miura et al., 2006). Nevertheless, other fish studies have shown contradictory results associated with ghrelin administration and food intake stimulation (Kang et al., 2011; Tinoco et al., 2014; Volkoff, 2016; Blanco et al., 2017). In our study, hypothalamic orexigenic neuropeptides and *ghsr* were upregulated by the fasting intervention, which is broadly consistent with the major trends observed in other fasting studies (Sainsbury and Zhang, 2010; Volkoff et al., 2010). However, the observed modulation of ghrelin was not consistent with expectations for the fasting challenge.

An absence of ghrelin mRNA upregulation under fasting intervention has been observed in other fish larval fasting study performed in Atlantic halibut (*Hippoglossus hippoglossus*) (Gomes et al., 2015), in agreement with our results. Additionally, metabolic studies carried out in genetic mouse models have shown that ghrelin is not a critical peptide for growth, maintenance of body weight or regulation of food intake (Al Massadi et al., 2015). However, it is important to consider that the fish larval stage is a special time point in a fish’s life characterized by metamorphosis (Rønnestad and Morais, 2008), a high growth potential (Opazo et al., 2017b), low vitelline reserves (Önal and Langdon, 2000), and high nutritional requirements (Hamre et al., 2013). Thus, orexigenic stimulation and regulation play a critical role in larval viability in nature.

The absence of differences in ghrelin between the groups may be explained by two possibilities: (i) the fasted group does not exhibit an increase in ghrelin mRNA levels or protein levels under fasting intervention; or conversely, (ii) the fed group does not exhibit a decrease in ghrelin mRNA levels and protein levels, due to absorbing feed nutrients (Yin et al., 2009). We think that the most feasible alternative to explain the study observations is the first possibility. Therefore, zebrafish larvae at 25 dpf are unable to increase their ghrelin levels (mRNA or protein), because they do not exhibit an adequate number of X/A-like cells to significantly increase the protein and mRNA levels of ghrelin. One element that supports this idea is that fish larvae exhibit a gradual increase in ghrelin gene expression associated with larval growth (Manning et al., 2008; Eom et al., 2013; Gomes et al., 2015); hence, ghrelin levels (mRNA or protein) are significantly correlated with larval body length. This correlation was observed in our study as well as in rat neonates (Hayashida et al., 2002). The second possibility referenced above may be explained by a special adaptation of fish larva X/A-like cells associated with ghrelin/nutrient regulation that could enhance the hypothalamic orexigenic state during the larval stage; however, this proposal regarding physiological regulation is only speculative and least likely.

Another outcome is associated with the upregulation of *npy* and *agrp* mRNA levels observed in fasting larvae, which suggests that the hypothalamus can sense energy homeostasis independent of the ghrelin signal. Because ghrelin is the only orexigenic peripheral peptide factor that has been described to date (Higgins et al., 2007), other factors could influence the orexigenic state associated with the upregulation of *npy* and *agrp*. The most likely factor could be the endocannabinoid system (ECS), which is a neuromodulatory system that plays an important role in food intake regulation (Jager and Witkamp, 2014; Volkoff et al., 2017).

Our future research prospects will address these possible larval food intake adaptations with more robust approaches and will address the role of the ECS in the peripheral control of food intake at the larval stage.

## Conclusion

In conclusion, a fasting intervention in zebrafish larvae resulted in significant increases in the mRNA levels of the orexigenic hypothalamic peptides *agrp, npy*, and *ghsr*. However, this upregulation in the fasted experimental group was not associated with an increase in ghrelin relative mRNA levels or ghrelin levels assessed via three different technical approaches. The modulation of ghrelin in zebrafish larvae was not consistent with expectations for the fasting intervention; therefore, ghrelin is not an essential peptide for appetite regulation in the zebrafish larval stage.

## Author Contributions

RO, FP-P, and LV conceived and designed experiments. RO and FP-P performed the experiments. RO, FP-P, GCdS, and GC performed the instrumental analyses of the study (gene expression, immunohistochemistry, or nLC-HRMS). RO, FP-P, GCdS, and VS performed the data analysis. RO, JR, GCdS, and VS wrote the manuscript. All authors read and approved the final version of the manuscript.

## Conflict of Interest Statement

The authors declare that the research was conducted in the absence of any commercial or financial relationships that could be construed as a potential conflict of interest.
